# Hematopoietic *Npc1* mutation shifts gut microbiota composition in *Ldlr*^−/−^ mice on a high-fat, high-cholesterol diet

**DOI:** 10.1038/s41598-019-51525-x

**Published:** 2019-10-18

**Authors:** Tom Houben, John Penders, Yvonne Oligschlaeger, Inês A. Magro dos Reis, Marc-Jan Bonder, Debby P. Koonen, Jingyuan Fu, Marten H. Hofker, Ronit Shiri-Sverdlov

**Affiliations:** 1 Departments of Molecular Genetics and Medical Microbiology, School of Nutrition and Translational Research in Metabolism (NUTRIM), Maastricht University, Maastricht, The Netherlands; 2 Department of Genetics, University Medical Center Groningen, University of Groningen, Groningen, The Netherlands; 3 Department of Pediatrics, Section Molecular Genetics, University Medical Center Groningen, University of Groningen, Groningen, The Netherlands

**Keywords:** Applied microbiology, Metabolic syndrome, Chronic inflammation

## Abstract

While the link between diet-induced changes in gut microbiota and lipid metabolism in metabolic syndrome (MetS) has been established, the contribution of host genetics is rather unexplored. As several findings suggested a role for the lysosomal lipid transporter Niemann-Pick type C1 (NPC1) in macrophages during MetS, we here explored whether a hematopoietic *Npc1* mutation, induced via bone marrow transplantation, influences gut microbiota composition in low-density lipoprotein receptor knockout (*Ldlr*^−/−^) mice fed a high-fat, high-cholesterol (HFC) diet for 12 weeks. *Ldlr*^−/−^ mice fed a HFC diet mimic a human plasma lipoprotein profile and show features of MetS, providing a model to explore the role of host genetics on gut microbiota under MetS conditions. Fecal samples were used to profile the microbial composition by 16 s ribosomal RNA gene sequencing. The hematopoietic *Npc1* mutation shifted the gut microbiota composition and increased microbial richness and diversity. Variations in plasma lipid levels correlated with microbial diversity and richness as well as with several bacterial genera. This study suggests that host genetic influences on lipid metabolism affect the gut microbiome under MetS conditions. Future research investigating the role of host genetics on gut microbiota might therefore lead to identification of diagnostic and therapeutic targets for MetS.

## Introduction

Metabolic syndrome (MetS) is a complex disorder that identifies centrally obese patients at increased risk for developing cardiovascular disease (CVD) and diabetes mellitus type 2^1,2^. Due to the continuous increased prevalence of obesity, MetS is considered a global epidemic, putting an enormous pressure on health care services^3^. Next to insulin resistance and hypertension, another central abnormality observed within MetS are disturbances in lipid metabolism, resulting in dyslipidemia^1,4^. Dyslipidemia is characterized by increased plasma triglyceride-rich lipoproteins, decreased plasma high-density lipoprotein (HDL) and increased plasma low-density lipoprotein (LDL), leading to MetS-associated pathologies^4^.

Next to disturbances in plasma lipid levels, alterations in intracellular lipid metabolism have also been observed in MetS. While mutations in the gene encoding for the lysosomal membrane protein Niemann-Pick type C1 (NPC1) are well-known to induce a rare lysosomal storage disease, recent findings have also linked a dysfunctional NPC1 protein to the development of obesity and MetS^5,6^. Specifically, heterozygous *Npc1* mutation carriers showed a 4.8 higher risk for developing morbid adult obesity^7^. Moreover, identification of several *Npc1* small nucleotide polymorphism (SNPs) via genome-wide association^5^ and confirmation studies^8–10^ have further highlighted the link between *Npc1* variants (allele frequency ~38%) and obesity in the European population. Mechanistically, a dysfunctional NPC1 protein leads to lysosomal lipid accumulation, thereby disturbing lipid metabolism^11^. Likewise, the phenomenon of lipid accumulation within the endo-lysosomal compartment of cells has also been observed in pathologies associated with MetS^12^. While lysosomal dysfunction in proximal tubular cells contributed to obesity-related kidney diseases^13^, lysosomal lipid storage within macrophages is a well-described mechanism disturbing general lipid metabolism and results in the induction of a chronic low-grade inflammatory response in non-alcoholic steatohepatitis^14,15^ and atherosclerosis^12,16–18^. These findings imply a role for macrophage NPC1 in disturbing lipid homeostasis in MetS.

Relevantly, several studies have shown a link between the composition of the gut microbiota and lipid parameters such as LDL, triglyceride and HDL levels^19–23^. Moreover, modulating the gut microbiota by transferring the microbiota of lean donors has been shown to improve MetS in patients^24^, indicating the gut microbiota as a promising target to tackle metabolic disorders. However, the underlying mechanisms through which the gut microbiota modulates host’s metabolism remain largely elusive^25^. Gaining knowledge into the exact interactions between environment and host genetics on gut microbiota and associated pathophysiological processes is therefore essential to improve our diagnostic and therapeutic approaches to tackle metabolic pathologies.

The current study investigated whether a hematopoietic *Npc1* mutation influences the gut microbiota in low-density lipoprotein receptor knockout (*Ldlr*^−/−^) mice fed a high-fat, high-cholesterol (HFC) diet for 12 weeks. *Ldlr*^−/−^ mice fed a HFC diet mimic a human plasma lipoprotein profile and show features of MetS^26^, providing a model to explore the role of host genetics on gut microbiota under MetS conditions. For this purpose, *Ldlr*^−/−^ mice received bone marrow from Niemann-Pick type C1 mutant (*Npc1*^*mut*^) mice. This study suggests that host genetic influences related to lipid metabolism shift the gut microbiota composition and increases microbial richness and diversity under MetS conditions. Variations in plasma lipid levels correlated with microbial diversity and richness as well as with several bacterial genera. Future research investigating the role of host genetics on gut microbiota can therefore lead to identification of novel diagnostic and therapeutic targets for MetS.

## Results

### Hematopoietic *Npc1* mutation disturbs intracellular and circulating lipid levels

**Figure 1:**
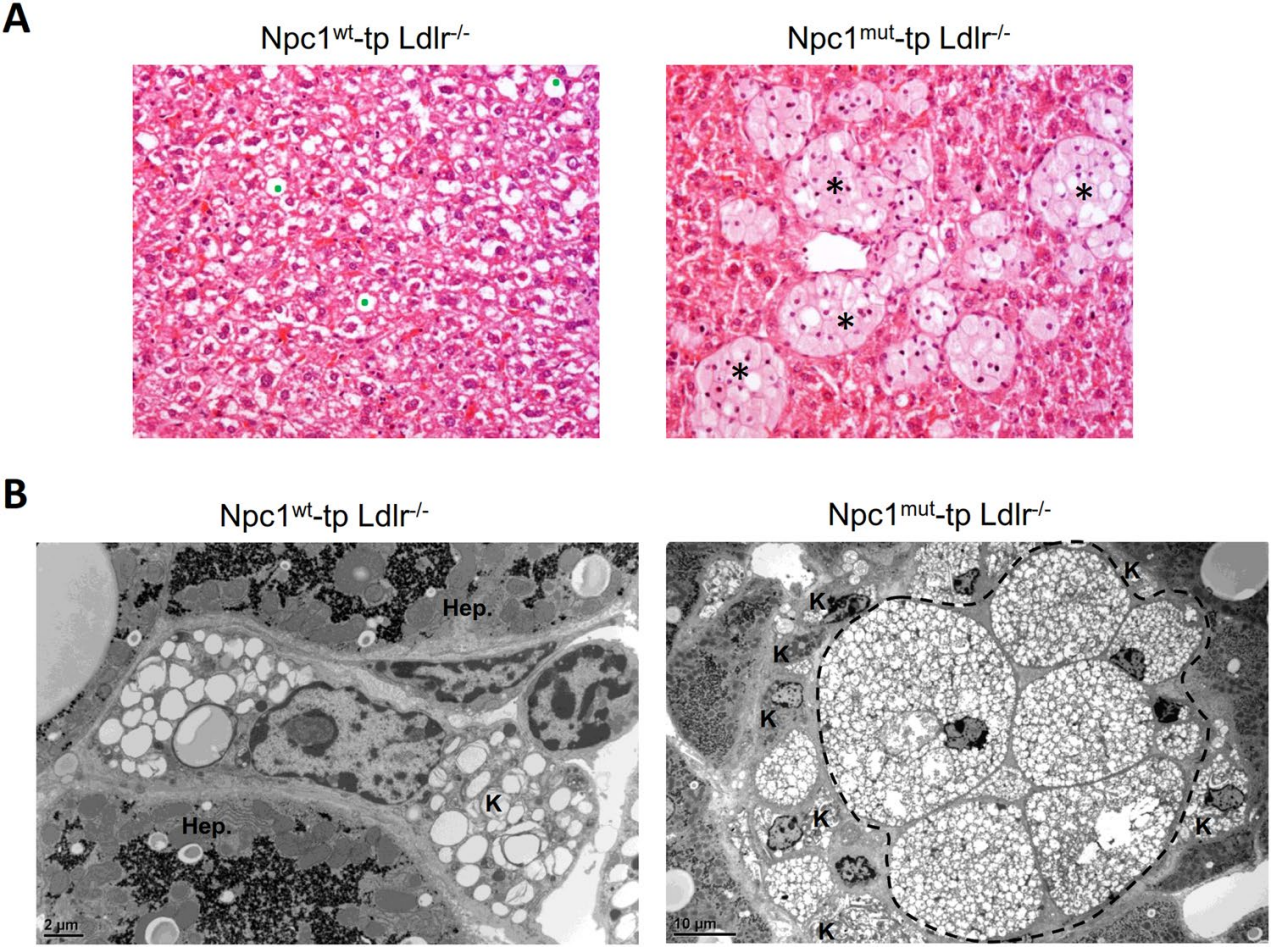
Hepatic phenotype of HFC-fed *Ldlr*^−/−^ mice carrying a hematopoietic *Npc1* mutation. (**A**) Representative images of hepatic staining for hematoxylin and eosin (H&E), showing lysosomal lipid accumulation and granuloma formation in *Npc1*^*mut*^-transplanted *Ldlr*^−/−^ mice. The left panel shows a normal view of the liver under high cholesterol conditions, with the green dots indicating lipid droplets. The right panel show clustering of foamy macrophages, referred to as granulomas (indicated by the asterisks). (**B**) Representative electron microscopy image of hepatic macrophages carrying a hematopoietic *Npc1* mutation in HFC-fed *Ldlr*^−/−^ mice, confirming the massive accumulation of lipids. The left panel shows a single Kupffer cell (K), surrounded by 2 hepatocytes (Hep.). The right panel shows the granuloma (surrounded by the dashed line), which is surrounded by Kupffer cells (K).

To confirm successfulness of the bone marrow transplantation, intracellular and circulating lipid levels were measured in *Npc1*^*mut*^-tp *Ldlr*^−/−^ mice. Subjecting *Npc1*^*mut*^-tp *Ldlr*^−/−^ mice to a HFC diet for 12 weeks resulted in a strong increase in hepatic cholesterol levels, while a reduction was observed for hepatic triglyceride levels (Table 1). Moreover, due to the hematopoietic *Npc1* mutation, hepatic lipids accumulated in the lysosomal fraction of hepatic macrophages (Fig. 1A,B and Supplementary Figs S1 and S2). Additionally, plasma cholesterol, triglyceride and free fatty acid levels decreased significantly, indicating a decrease of circulating lipids in *Npc1*^*mut*^-tp *Ldlr*^−/−^ mice (Table 1). In line with these data, hepatic gene expression levels of ATP-binding cassette sub-family G member 1(*Abcg1*), Niemann-Pick type C2 (*Npc2*) and ATP-binding cassette transporter A1 (*Abca1*) increased in *Npc1*^*mut*^-tp *Ldlr*^−/−^ mice. In spite of increased hepatic cholesterol levels (Table 1), these observations indicate increased hepatic cholesterol efflux signaling, which is likely a compensation mechanism to overcome the accumulation of cholesterol in lysosomes. Overall, these data show that a hematopoietic *Npc1* mutation induces lysosomal accumulation of lipids inside liver macrophages and disturbs general lipid metabolism in *Ldlr*^−/−^ mice on a HFC diet.

**Table 1:** Intracellular and circulating lipid metabolism.

	Npc1^wt^-tp Ldlr^−/−^	Npc1^mut^-tp Ldlr^−/−^
Liver total cholesterol (µg/mg dry weight)	47.03 ± 4.47	75.47 ± 2.51***
Liver total triglycerides (mg/mg protein)	0.51 ± 0.06	0.26 ± 0.04**
Plasma total cholesterol (mM)	38.53 ± 1.44	22.1 ± 1.09***
Plasma total triglycerides (mM)	3.82 ± 0.31	1.42 ± 0.15***
Plasma free fatty acids (mM)	1.33 ± 0.04	0.96 ± 0.05***
*Abcg1 (Rel. Expr.)*	1.00 ± 0.09	2.65 ± 0.13***
*Npc2 (Rel. Expr.)*	1.00 ± 0.1	3.06 ± 0.12***
*Abca1 (Rel. Expr.)*	1.00 ± 0.12	1.13 ± 0.06

### Microbial richness and diversity are increased and linked to lipid metabolism

**Figure 2:**
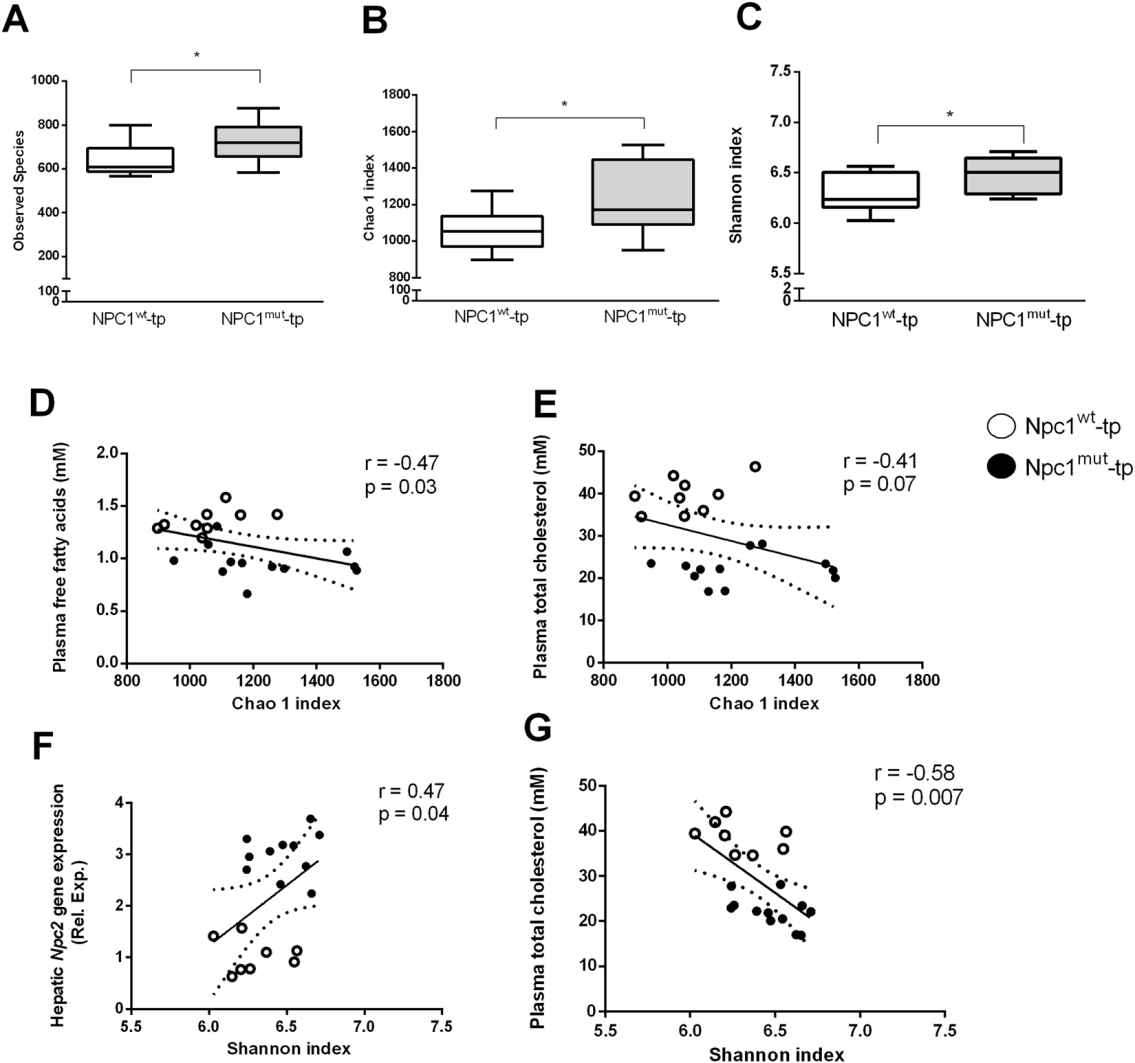
Alpha diversity metrics in *Ldlr*^−/−^ mice on HFC diet carrying a hematopoietic *Npc*1 mutation compared to wildtype. (**A**–**C**) The number of OTUs (**A**), Chao 1 index (**B**) and Shannon index (**C**) indicate increased diversity in *Npc1*^*mut*^-transplanted *Ldlr*^−/−^ mice. (**D**–**G**) Spearman correlation between alpha diversity metrics and lipid parameters indicate a link between lipid metabolism and gut microbiota diversity. ^*^Indicates p < 0.05. All errors are SEM.

To investigate the influence on microbial richness and diversity, α-diversity metrics were calculated. Both the observed microbial richness (observed number of OTUs) as well as the estimated richness as indicated by the Chao 1 index, was significantly higher in *Ldlr*^−/−^ mice carrying the hematopoietic *Npc1* mutation (Fig. 2A,B). Likewise, microbial biodiversity, measured via the Shannon index (Fig. 2C) was significantly increased in *Npc1*^*mut*^-tp mice. To further define which variables are linked to microbial richness and diversity in this mouse model, Spearman correlation analysis was performed on all variables and the Chao 1 (richness) or Shannon (biodiversity) index. Plasma free fatty acids levels were negatively correlated with microbial richness (r = −0.47; p = 0.03) (Fig. 2D). Concomitantly, plasma total cholesterol levels showed the same inverse correlation with microbial richness (r = −0.41; p = 0.07) (Fig. 2E), though this correlation did not reach statistical significance. Furthermore, biodiversity correlated positively with hepatic gene expression levels of the intracellular cholesterol transporter Niemann-Pick type C2 (*Npc2*) (r = 0.47; p = 0.04) (Fig. 2F) and negatively with plasma total cholesterol levels (r = −0.56; p = 0.007) (Fig. 2G). Together, these data demonstrate that a hematopoietic *Npc1* mutation in *Ldlr*^−/−^ mice increases microbial richness and biodiversity, which is correlated to changes in lipid metabolism.

### The microbial community structure is related to the hematopoietic *Npc1* mutation but the latter is not linked to enterotypes

**Figure 3:**
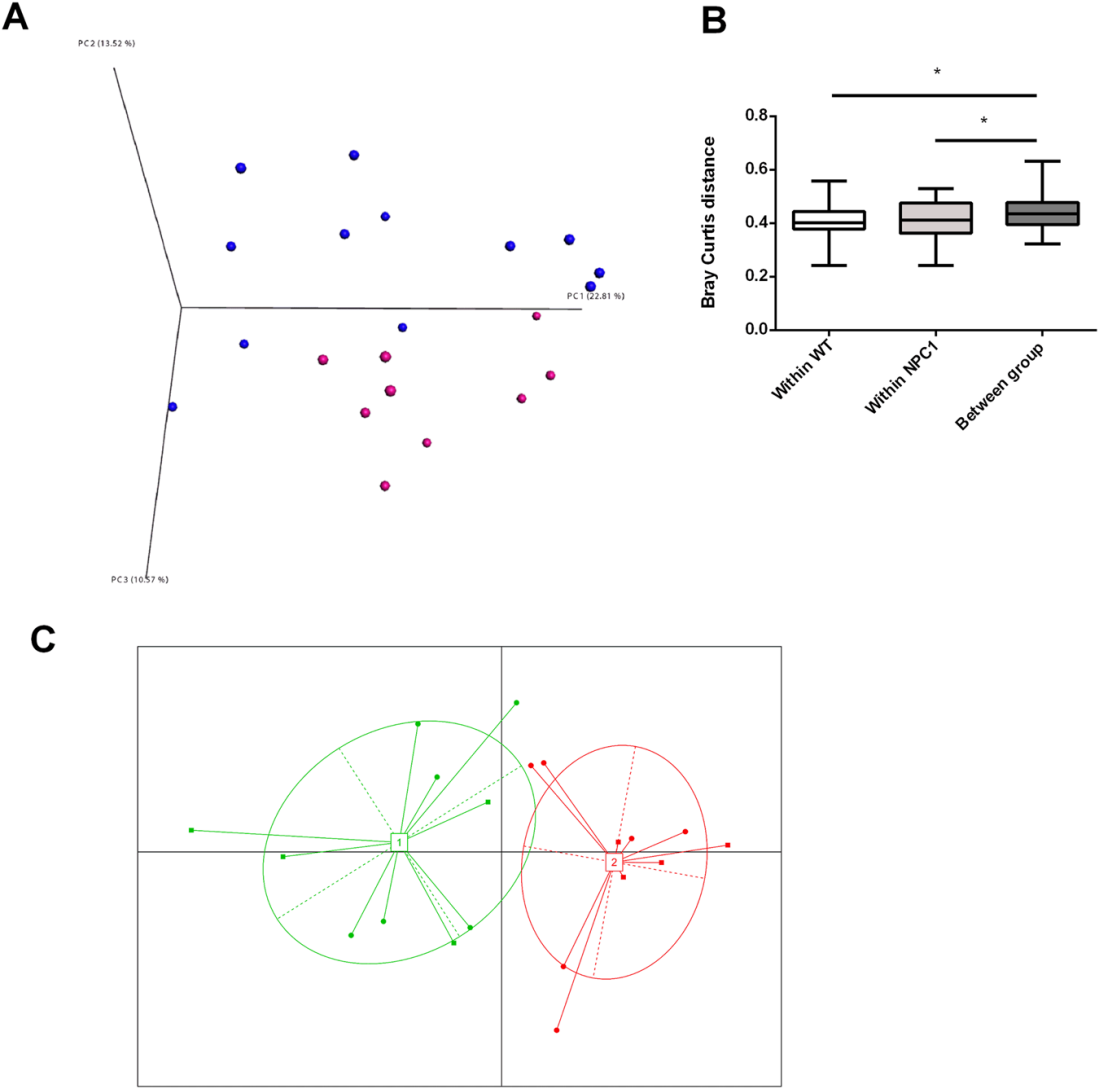
Principal Coordinate Analyses (PCoAs) based on Bray-Curtis distance and assortment of gut microbial communities into enterotypes for HFC-fed *Ldlr*^−/−^ mice carrying a hematopoietic *Npc1*^*wt*^ or *Npc1*^*mut*^ mutation. (**A**) PCoA-plot based on unweighted UniFrac metrics for all samples showed that samples could be partly separated based upon the hematopoietic Npc1 mutation. (**B**) Bray-Curtis distances were calculated within *Npc1*^*wt*^-transplanted and *Npc1*^*mut*^-transplanted HFC-fed *Ldlr*^−/−^ mice (average pairwise distance in microbiota composition between samples of the same experimental group) and between *Npc1*^*wt*^ -transplanted and *Npc1*^*mut*^ -transplanted HFC-fed *Ldlr*^−/−^ mice (average pairwise distance between samples of different experimental groups), confirming the separation based on the hematopoietic Npc1 mutation. (**C**) Between-class analysis visualizing the two distinct enterotypes that could be identified based upon PAM clustering of Bray-Curtis distances, with closed dots (*Npc1*^*mu****t***^) and squares (*Npc1*^*w****t***^) representing individual mice and numbered white rectangles marking the center of each enterotype. Enterotypes were mainly driven by differential abundances in *Allobaculum*, *Lactobacillus* and *S24-7*, however the clustering was not associated with the bone marrow-specific *Npc1* mutation. *Indicates p < 0.05.

The dissimilarity in the microbial community composition (beta-diversity) of stool samples was assessed using the Bray-Curtis (BC) dissimilarities. Principal coordinate analysis (PCoA) based on Bray-Curtis dissimilarities showed that samples could be partly separated based upon the hematopoietic Npc1 mutation (Fig. 3A). Moreover, the within-group distance in microbial community structure (i.e. average Bray-Curtis dissimilarity between samples from the same treatment group) was significantly smaller than the between-group distance, indicating that the microbiota composition of animals within the same treatment group is more similar compared to animals from different treatment groups (Fig. 3B). Enterotype analyses revealed two enterotypes driven by Allobaculum, Lactobacillus and S24-7 (Fig. 3C). However, the clustering was not associated with the bone marrow-specific *Npc1* mutation.

**Figure 4:**
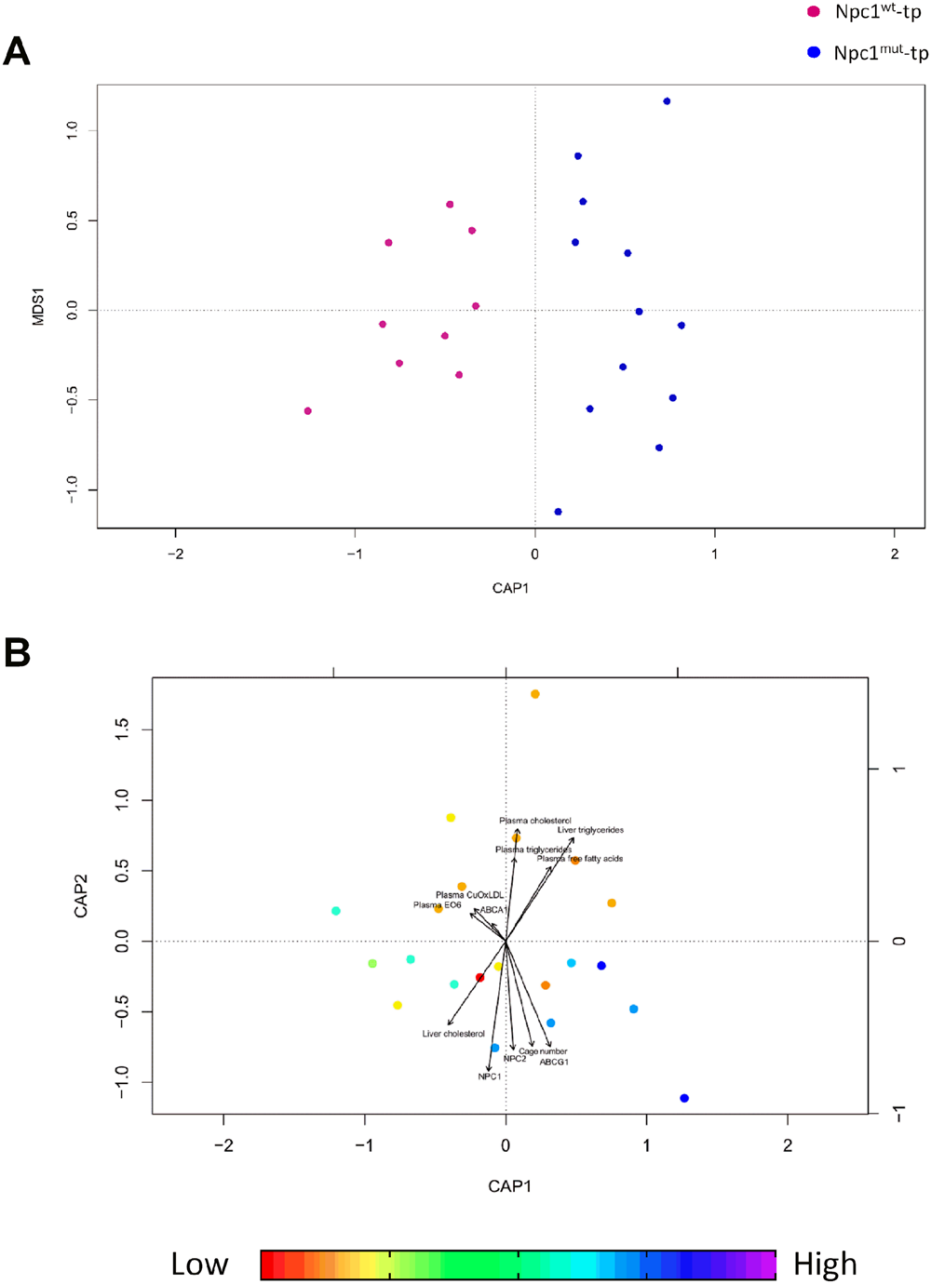
Distance-based Redundancy Analysis (db-RDA) showing the relationship of the hematopoietic deficiency for *Npc1* and *Abcg1* to the microbial community structure. The plots represent a dbRDA ordination based upon the Bray-Curtis distance including (**A**) *Npc1*^*wt*^- and *Npc1*^*mut*^ -transplanted *Ldlr*^−/−^ mice with the hematopoietic Npc1 mutation as explanatory variable. This panel confirms the separation of the microbiota based on the hematopoietic Npc1 mutation. (**B**) Plasma cholesterol, plasma triglycerides, liver triglycerides, plasma free fatty acids, cage number, liver cholesterol, plasma EO6, plasma CuOxLDL and hepatic gene expression of *Abcg1*, *Npc2*, *Npc1* and *Abca1* as explanatory variables and samples colored to hepatic expression of *Abcg1*. Multivariate PERMANOVA analysis including all explanatory variables demonstrated that both *Npc1* and *Abcg1* were significantly associated with the microbial community structure, while all other explanatory variables were not when mutually adjusting for each other. This plot further confirms that increased *Npc1* and *Abcg1* expression levels are associated with similar changes in the microbial community structure. MDS1 represents the unconstrained axis. CAP1 and CAP2 represent, respectively, the first and second constrained axes used in the CAP (canonical analysis of principal coordinates).

To determine whether the hematopoietic *Npc1* mutation or other variables had an additional effect on the gut microbiota community structure, we subsequently performed distance-based redundancy analysis (dbRDA); a constrained ordination technique. Constrained ordination techniques attempt to explain differences in microbial composition between samples by differences in explanatory variables (e.g. hematopoietic *Npc1* mutation). In db-RDA, the information from explanatory variables is combined with the eigenvalues obtained from the PCoA. Permutational multivariate analysis of variance (PERMANOVA) was used to determine significant dissimilarity within bacterial communities and multiple explanatory variables. The hematopoietic *Npc1* mutation (p = 0.034, explained variance = 18.9%) as well as hepatic gene expression levels of the cholesterol transporter *Abcg1* (p = 0.041, explained variance = 20.8%) showed a relationship with the microbial community structure (Fig. 4A,B), whereas *Abca1*, *Npc2*, plasma total cholesterol, plasma total triglycerides, plasma free fatty acids, plasma cupper oxLDL, plasma EO6, cage number, liver total cholesterol and liver total triglycerides did not.

### Hematopoietic *Npc1* mutation shifts gut microbiota composition in HFC-fed *Ldlr*^−/−^ mice

**Figure 5:**
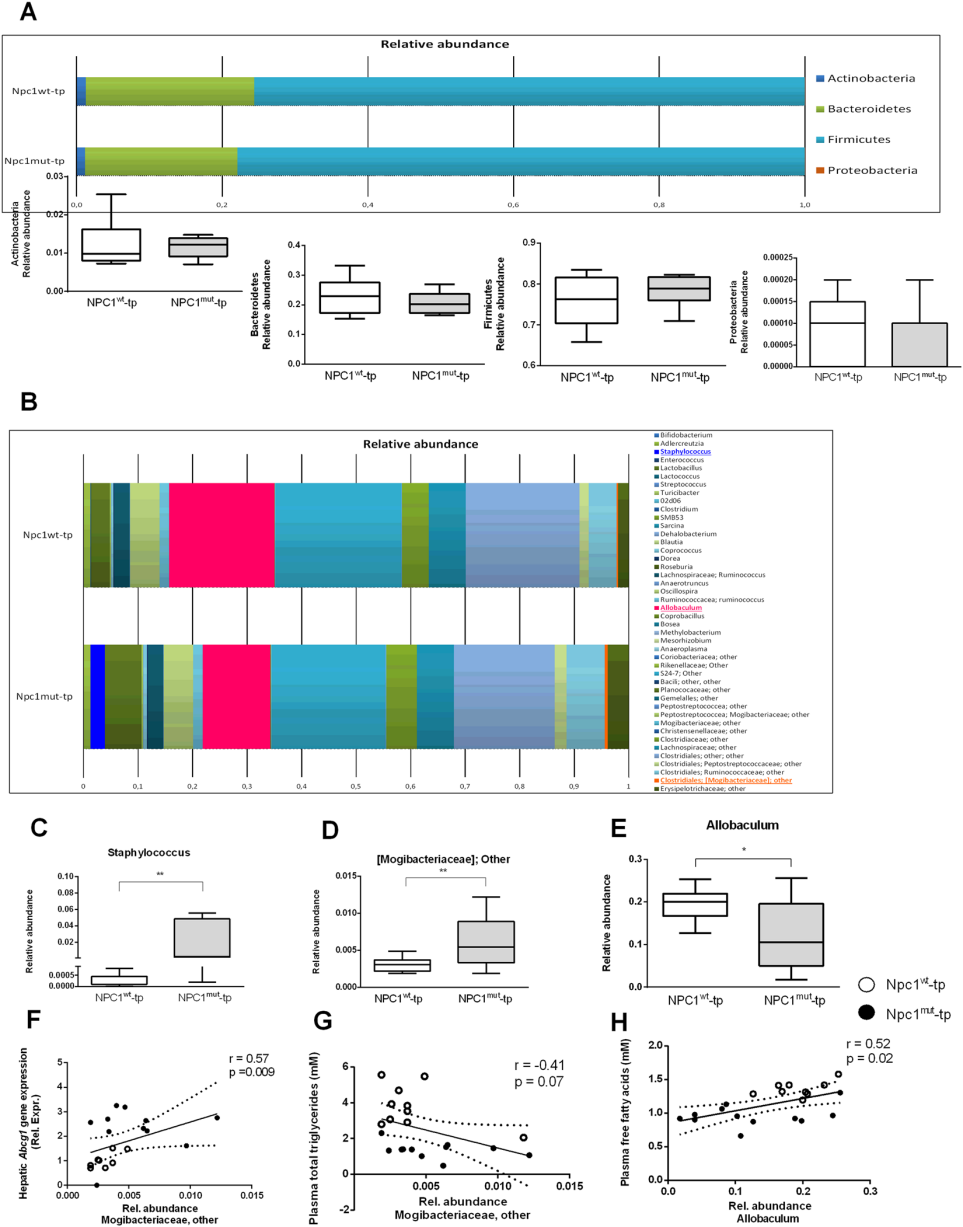
Taxonomy at phylum and genus level for *Npc1*^*wt*^*-* and *Npc1*^*mut*^ -transplanted HFC-fed *Ldlr*^−/−^ mice. (**A**,**B**) Overview of the mean relative abundance at phylum level. (**C**) An overview of the mean relative abundance at genus level is given. Significant differences between the groups at genus level are presented in separate box-whiskers for *Staphylococcus spp*. (**D**), unclassified *Mogibacteriaceae spp*. (**E**) and *Allobaculum spp*. (**F**). (**G**–**I**) Spearman correlation between lipid parameters and relative abundances of unclassified *Mogibacteriaceae spp*. and *Allobaculum spp*. ^*^Indicates p < 0.05 and ^**^ p < 0.01.

Based on 16S rRNA gene sequencing, the dominant phyla/genera were selected for further analysis, leading to in depth analysis of 4 phyla and 44 genera (Fig. 5A,B). Firmicutes, Bacteroidetes and, to a lesser extent, Actinobacteria and Proteobacteria were the dominant observed phyla (Fig. 5A). The relative abundance of the bacterial phyla was not significantly different between *Npc1*^*wt*^- and *Npc1*^*mut*^-tp mice (Fig. 5A), however, within the Firmicutes phylum significant differences were observed in the relative abundance of several bacterial genera. Specifically, *Staphylococcus spp*. (p = 0.008; q = 0.34) and unclassified *Mogibacteriaceae spp*. (p = 0.008; q = 0.34) showed significantly higher levels in relative abundance in the *Npc1*^*mut*^-tp mice compared to *Npc1*^*wt*^-tp mice, whereas the abundance of *Allobaculum spp*. (p = 0.01, q = 0.34) was significantly lower (Fig. 5B–E). Relevantly, the unclassified *Mogibacteriaceae spp*. correlated with hepatic gene expression levels of *Abcg1* (r = 0.57; p = 0.009) (Fig. 5F) and plasma total triglyceride levels (r = −0.41; p = 0.07), though the latter association was not statistically significant (Fig. 5G). Furthermore, the genus *Allobaculum spp*. showed a positive correlation with plasma free fatty acid levels (r = 0.52; p = 0.02) (Fig. 5H). Thus, a hematopoietic *Npc1* mutation alters the composition of gut microbiota in HFC-fed *Ldlr*^−/−^ mice, which are correlated with changes in lipid metabolism.

## Discussion

Whereas increasing evidence has linked diet-induced changes in gut microbiota to perturbations in lipid metabolism in MetS, the contribution of host genetics to this relation is rather unexplored. This study suggests that host genetic influences on lipid metabolism such as a hematopoietic *Npc1* mutation affect the gut microbiome under MetS conditions.

Though *Npc1* was previously identified in a human GWAS for obesity^5^, our study is the first to imply its direct impact on the gut microbiome under MetS conditions. This observation suggests that host-derived genetic disturbances in lipid metabolism can impact the gut microbiome. Indeed, an increasing amount of reports have established the capacity of the host’s genetic profile to shape the gut microbiome, thereby supporting our findings^27,28^. Also, the observation that a hematopoietic *Npc1* mutation impacts the gut microbiota under MetS conditions confirms the previously established link between lipid metabolism and gut microbiota composition. However, in contrast to genetic manipulation of lipid metabolism, the link between lipid metabolism and gut microbiota has mainly been established based on studies wherein dietary components (i.e. high fat diet, prebiotics) altered lipid metabolism via influences on gut microbiota^29,30^. Therefore, in addition to gut microbiota-mediated influences of dietary compounds on lipid metabolism, our study implies that also host genetic factors that influence lipid metabolism can impact the gut microbiome. However, as we employed a HFC diet to both experimental conditions, the observed impact of the gut microbiota of the *Npc1* mutation is likely also influenced by this diet. Indeed, previous reports have indicated that a high fat diet influences the composition of the gut microbiota^31,32^. Therefore, in spite of the observed changes in gut microbiota composition, to incontrovertibly demonstrate that the hematopoietic *Npc1* mutation influences gut microbiota composition, it is essential to investigate the gut microbiota of *Npc1*^*mut*^–tp *Ldlr*^−/−^ mice receiving a normal chow diet.

Our results also shed new light on the association between overall health status and the gut bacterial richness and diversity. Here, under HFC conditions, induction of the pathological hematopoietic *Npc1* mutation elevated bacterial richness and diversity. However, this is opposed to the concept that increased bacterial richness and diversity reflect ecosystem stability and resilience and is associated with overall health^33–37^. Yet, our apparent discordant finding on the association between disease and richness/diversity has also been observed by Kasai *et al*. who observed increased levels of bacterial diversity in obese compared to lean subjects^38^. Moreover, Vandeputte *et al*. associated stool consistency, potentially due to long colonic transit time^39,40^, with higher gut bacterial richness. Therefore, the increased bacterial richness and diversity observed in the pathological experimental group in the current study might have been influenced by stool consistency, hampering the view on bacterial richness and diversity as a marker for overall health status. Of note, while it is generally accepted that reduced plasma lipids are beneficial for health^41^, in the current model the reduced plasma lipids are a consequence of the accumulation of lipids inside macrophages (due to the *Npc1* mutation). Therefore, the observed reduction in plasma lipids in our model is not considered beneficial for the health status, as these mice develop inflammation in the liver^42^ and arteries^43^.

Among the shifted gut bacteria after hematopoietic *Npc1* mutation, we identified reduced relative abundances of the genus *Allobaculum spp*. Several studies have previously shown that *Allobaculum spp*. abundance is reduced in response to dietary fat intake^44,45^ and correlates with variations in plasma HDL concentrations^46^ and body weight^47^. In line, hepatic *Abcg1* and *Abca1* expression was increased in *Npc1*^*mut*^-tp mice, suggesting changes in lipid metabolism via lysosomal lipid-mediated activation of the transcription factor liver X receptor^48,49^. Therefore, our finding is consistent with the proposed view that *Allobaculum spp*. can be considered beneficial for the physiology of the host^29^. Next, in a prospective cross-sectional study, *Staphylococcus spp*. abundance was positively correlated with energy intake in obese children^50^. Here, we found an increased abundance of *Staphylococcus spp*. in HFC-fed *Ldlr*^−/−^ mice carrying the hematopoietic *Npc1* mutation. As such, *Staphylococcus spp*. might be involved in regulating the host’s lipid metabolism. Finally, increased relative abundances were observed of unclassified *Mogibacteriaceae spp*. in this study. Relevantly, a recent study by Harach *et al*. correlated levels of unclassified *Mogibacteriaceae spp*. with amyloid beta 42 levels in the brain of mice suffering from Alzheimer’s disease^51^. As multiple reports have previously linked a dysfunctional NPC1 protein to Alzheimer’s disease^52–54^, relative abundance changes in unclassified *Mogibacteriaceae spp*. might be directly related to mutations in *Npc1* and deserve further investigation. Of note, to ensure the causality between mutations in *Npc1* and changes in gut microbiota composition, host responses should also be investigated in the future by means of fecal transplantation experiments. As such, the results in the current study do not provide information on causality, but are rather of associative nature.

Overall, this study suggests the impact of host genetic influences on lipid metabolism such as a hematopoietic *Npc1* mutation on the gut microbiome in the context of MetS, confirming the link between lipid metabolism and gut microbiota. In addition, in contrast to previous studies, our findings suggest that increased microbial richness and diversity do not necessarily associate with improved health. Despite the significant associations between microbial richness and diversity and several lipid parameters, these Spearman correlations were rather low, potentially due to low sample size. As such, further research is necessary to confirm these associations. Finally, future research investigating the role of host genetics on gut microbiota is therefore warranted as it will increase our understanding of the function of the gut microbiome in human physiology and pathology.

## Methods

### Experimental design

Niemann-Pick type C1^nih^ mutant (*Npc1*^*mut*^) mice (a kind gift from Prof. Dr. Lieberman from University of Michigan Medical School) were backcrossed into a C57BL/6J background for more than 10 generations. *Npc1*^*mut*^ and *Ldlr*^−/−^ mice were housed under standard conditions and had access to food and water ad libitum. Experiments were performed according to Dutch regulations and approved by the Committee for Animal Welfare of Maastricht University (approval number DEC 2011-133)^42,43^. Specifically, mice were housed in individually ventilated cages (IVC) in a conventional specified pathogenic free (SPF) facility where a 12 hr day/night cycle was handled using a dusk transition phase. Room temperature was kept constant at 21 °C. Corncob bedding material was used in the cages and animals were fed 10 mm food pellets that were irradiated with 25 kGray.

To generate myeloid *Npc1*^*mut*^ deficient *Ldlr*^−/−^ mice, a bone marrow transplantation was performed. Twenty-two week-old female *Ldlr*^−/−^ mice received one week before and four weeks after irradiation antibiotic water containing 100 mg/l neomycin (Gibco, Breda, the Netherlands) and 6 * 10^4^ U/l polymycin B Sulphate (Gibco, Breda, the Netherlands). One day before and on the day of the transplantation, *Ldlr*^−/−^ mice were lethally irradiated with 6 Gray of γ-radiation in the morning, thus receiving 12 Gray in total. Lethally irradiated *Ldlr*^−/−^ mice were then injected with 1 * 10^7^ bone marrow cells donated from either *Npc1*^*mut*^ mice or wildtype littermate controls (*Npc1*^*wt*^). In order to fully ensure bone marrow replacement, mice had a nine-week recovery period. After nine weeks of recovery, transplanted (-tp) mice received an HFC diet, containing 21% butter, 0.2% cholesterol, 46% carbohydrates and 17% casein (diet 1635; Scientific Animal Food and Engineering, Villemoissonsur-Orge, France) for 12 weeks. We further highlight that the same mouse model as well as the described lipid measurements were used in previously published manuscripts, where administration of dietary cholesterol was essential to induce lysosomal lipid-induced inflammation^42,43,55^. As we anticipated only subtle changes^56^ in the gut microbiome due to the *Npc1* mutation being restricted to the hematopoietic line, we did not cohouse both genotypes. Though the influence of cage-effects cannot be completely excluded, the following measures were taken to minimize the impact of housing conditions: the litters were separated per genotype after weaning, mice of similar age were used, the entire experiment was performed at once (so not over different time points), food and autoclaving parameters were standardized, only 1 person handled the mice throughout the entire experiment, mice were kept in cages in the same room, in the same rack and finally, mice of each genotype were put in four different cages. Finally, the between-genotype Bray-Curtis dissimilarity was significantly increased to the between-cage Bray-Curtis dissimilarity ((p = 0.03 compared to WT cages and p = 0.04 compared to *Npc1*^*mut*^ cages), providing further evidence that the observed effects in the current study are at least partly due to the *Npc1* mutation. Liver tissue was isolated and snap-frozen in liquid nitrogen and stored at −80 °C or fixed in 4% formaldehyde/PBS. The biochemical determination of plasma cholesterol and liver triglyceride levels, electron microscopy, RNA isolation, complementary DNA synthesis, quantitative polymerase chain reaction are described extensively^42,43,57^. Liver cholesterol levels were quantified as described previously^58^.

### Sample collection and processing

Mice were housed in groups of 4 in separate cages, which were kept in the same room throughout the study. Fecal samples were snap-frozen with liquid nitrogen and stored at −80 °C. DNA isolation was done using the FavorPrep Stool DNA Isolation Mini Kit (FASTI 001-1) according to manufacturer’s protocol.

### Sequencing

Amplicon libraries and sequencing was performed according to previously published protocols^19,59,60^. Briefly, the hypervariable region V4 of the 16S rRNA gene was PCR amplified from each DNA sample using forward primer 515 F and reverse primer 806 R as described previously^19^. Illumina MiSeq paired end sequencing of equimorlarly pooled amplicons was subsequently used to determine the bacterial composition of fecal samples. Custom scripts were used to remove primer sequences, align paired end reads and quality-filter the sequencing reads. Details can be found in Fu *et al*.^19^ and Bonder *et al*.^59^. Sequencing data of all samples have been deposited in the European Bioinformatics Institute (EBI) database under primary accession code (ENA) PRJEB31646 and secondary accession code ERP114028.

### Data analysis and statistics

Sequences were clustered into Operational Taxonomic Units (OTUs) using UCLUST (version 1.2.22q) at 97% similarity. For taxonomic assignments of OTUs, a TaxMan^61^ filtered and truncated version of the full Greengenes reference database version 13.5 was used. OTUs that did not cluster against any of the sequences in the reference database were suppressed.

A total of 1,503,804 paired-end sequences, with a sequencing depth ranging from 15,637–117,585 reads/sample, were clustered in a total of 13,312 OTUs. To further reduce spurious OTUs and normalize for sequencing depth, data were rarefied to 10,000 reads/sample and OTUs containing less than 5 reads or occurring in only a single sample were discarded, resulting in a final number of 2.695 OTUs. Downstream analysis were conducted in QIIME version 1.9^62^ and R version 3.1.3.

### Microbial data-analyses and statistics

#### Taxonomic composition

Differences in the relative abundance of bacterial phyla and genera between *Npc1*^*wt*^-tp and *Npc1*^*mut*^-tp *Ldlr*^−/−^ mice were compared using the non-parametric t-test Metastats while controlling for multiple comparisons by means of the False Discovery Rate (q = 0.15; 750 permutations). Relations between bacterial phyla/genera and other continuous variables were analyzed via Spearman correlation using GraphPad Prism, version 6.0 for Windows. Though correlations between lipid parameters and gut microbiota abundances were calculated on pooled data to increase the power, it was verified whether both groups showed the same effect.

#### Microbial richness & diversity and microbial community structure

The following metrics of species richness and diversity within communities (alpha-diversity) were determined: observed OTUs (observed richness), Chao1 index (estimated richness), and Shannon diversity index. Alpha diversity metrics between the wild-type and mutant animals were compared by Mann Whitney tests using GraphPad Prism, version 6.0 for Windows. Beta-diversity, or diversity shared across samples was determined by the Bray-Curtis dissimilarity (BC) at a rarefaction depth of 10,000 seq/sample (Supplementary Fig. S3). Clustering of samples was visualized using Principal Coordinate analysis (PCoA) followed by distance-based redundancy analysis (db-RDA), a constrained extension of PCoA. Db-RDA was performed in R^63^ package vegan using the capscale function. To determine to what extend the hematopoietic *Npc1* mutation contributed in explaining the microbial community structure, we used variance partitioning with distance-based redundancy analysis (db-RDA) of Bray-Curtis distances. In a subsequent db-RDA, the following data were additionally included as explanatory variables to examine whether they had a significant impact on the microbial community structure: liver cholesterol, liver triglycerides, plasma cholesterol, plasma triglycerides, plasma free fatty acids, plasma cupper oxLDL, plasma EO6, cage number and hepatic gene expression of *Abcg1*, *Npc2* and *Abca1*.

#### Enterotyping analyses

Enterotype analyses were performed as described previously by Armougham and colleagues^64^. Bray-Curtis (BC) distances were calculated for the genus-level relative abundance profiles. We used the R^63^ package “vegan: Community ecology”, version 2.2-1 by Oksanen *et al*. from 2011 for calculating the Bray-Curtis distances^65^.

To cluster the samples based on these distance metrics, we used the Partitioning Around Medoids (PAM) clustering algorithm in the R package “Cluster analysis basics and extensions”, version 2.0.1 ed. by Maechler *et al*. from 2012^66^. The optimal number of clusters was chosen based on Calinski-Harabasz (CH) index and validated by the silhouette index^67^ using the R package ‘clusterSim’.

An optimal number of 2 clusters was identified based on the CH index, which was confirmed by the silhouette index although clustering was weak (SI 0.28).

Subsequent visualization and identification of relevant taxa was conducted for analyses based on BC distance. Between-class analysis (BCA) was performed to plot the samples using the R package “Analysis of Ecological Data: Exploratory and Euclidean Methods in Environmental Sciences” version 1.7.2. by Dray *et al*. from 2015. The similarity percentage analysis (SIMPER)^68^ was used to identify taxa contributing to similarity within- and dissimilarity between groups. Similar analyses have also been described previously^60^.

## Supplementary information

Supplementary Info
