# Supplementary material for: Hematopoietic Npc1 mutation shifts gut microbiota composition in Ldlr−/− mice on a high-fat, high-cholesterol diet
Source: Sci Rep. 2019 Oct 18;9:14956. doi: 10.1038/s41598-019-51525-x (PMC6802207; doi:10.1038/s41598-019-51525-x)
Supplement: Supplementary file 1 — Supplementary Info [file 41598_2019_51525_MOESM1_ESM.pdf]

<u>Supplementary information</u>

Hematopoietic *Npc1* mutation shifts gut microbiota composition in *Ldlr*$^{-/-}$ mice on a high-fat, high-cholesterol diet.

Tom Houben, John Penders, Yvonne Oligschlaeger, Inês A. Magro dos Reis, Marc-Jan Bonder, Debby P Koonen, Jingyuan Fu, Marten H Hofker, Ronit Shiri-Sverdlov

Supplementary Figure S1

Npc1^{wt}-tp Ldlr$^{-/-}$ Npc1^{mut}-tp Ldlr$^{-/-}$

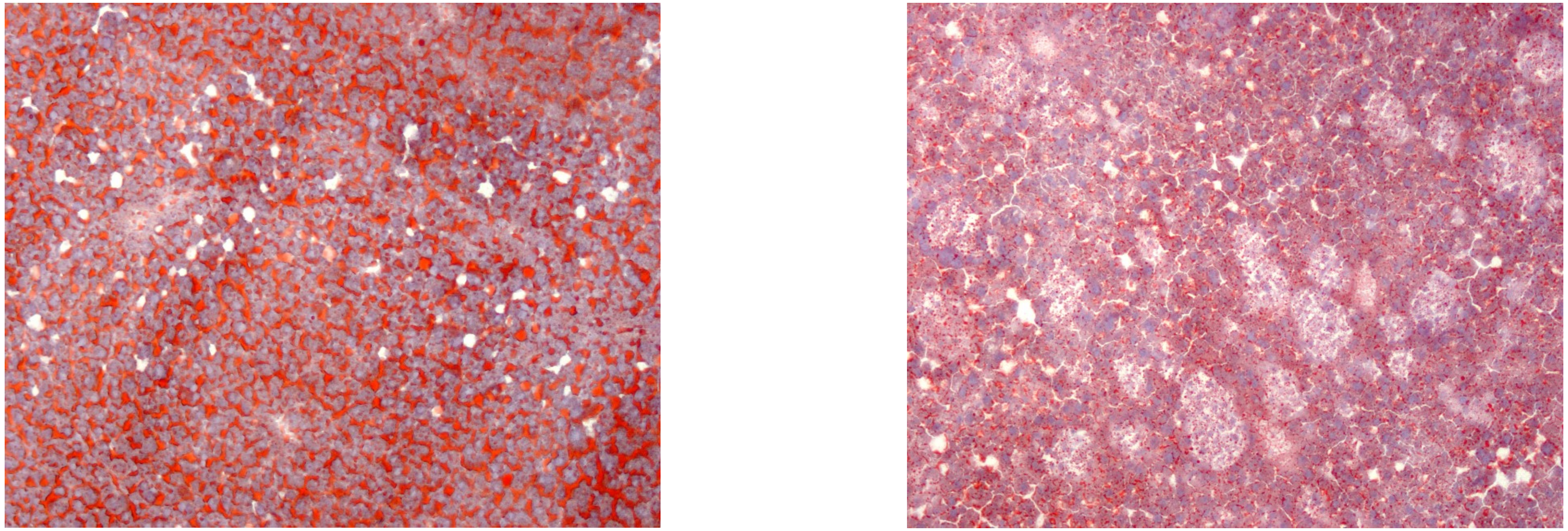

Supplementary Figure S1: Oil Red O staining

Supplementary Figure S2

Npc1^{wt}-tp Ldlr$^{-/-}$

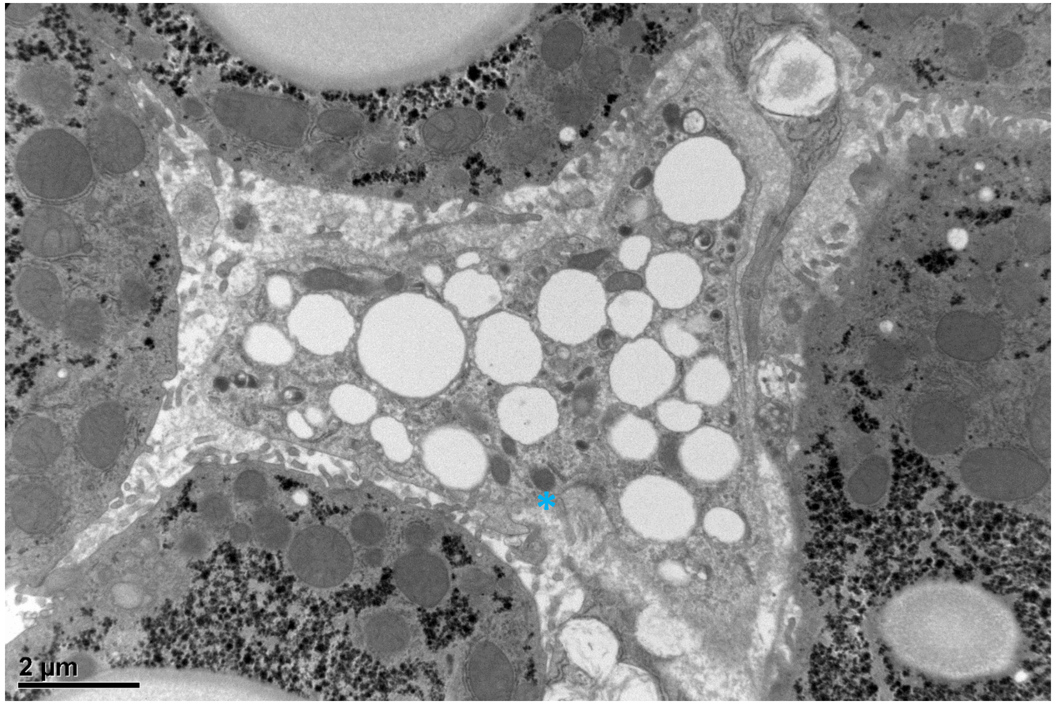

Npc1^{mut}-tp Ldlr$^{-/-}$

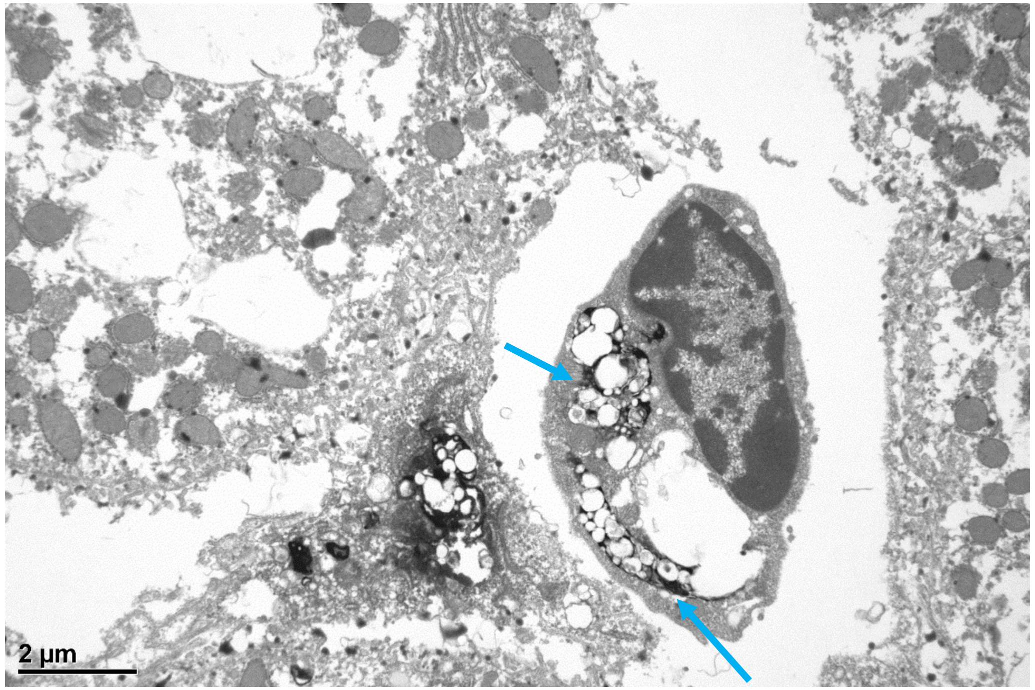

Supplementary Figure S2: Representative images of electron microscope wherein acid phosphatase staining was used for visualisation of lysosomes (blue asterisk and arrows) in hepatic macrophages

Supplementary Figure S3

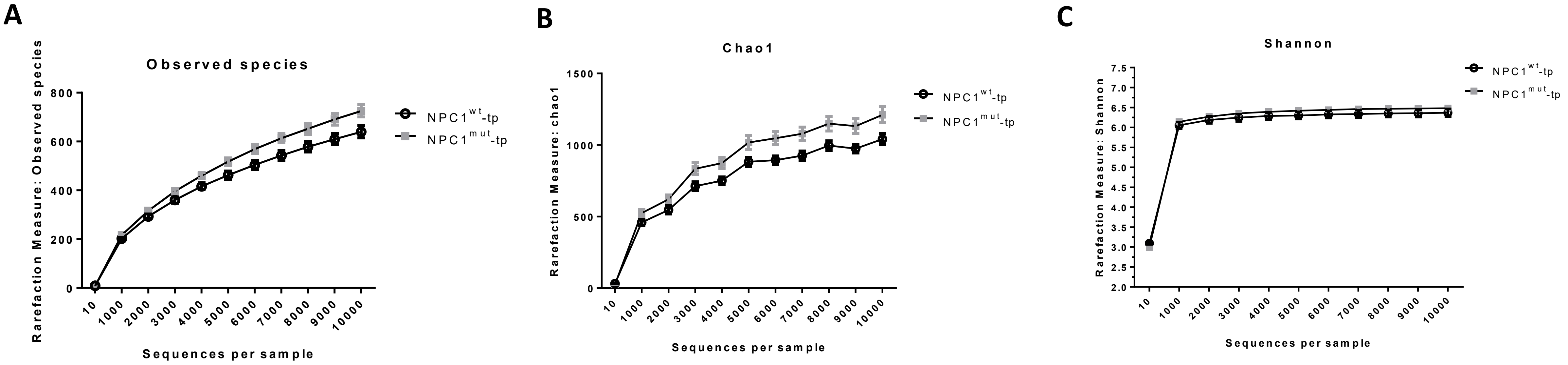

Supplementary Figure S3: Rarefaction plots
